# Diet Diversity and Micronutrient Adequacy among Filipino School-Age Children

**DOI:** 10.3390/nu11092197

**Published:** 2019-09-12

**Authors:** Tsz-Ning Mak, Imelda Angeles-Agdeppa, Yvonne M. Lenighan, Mario V. Capanzana, Ivan Montoliu

**Affiliations:** 1 Nestlé Research, Route du Jorat 57, 1000 Lausanne-26, Switzerland; Yvonne.Lenighan@rd.nestle.com (Y.M.L.); Ivan.MontoliuRoura@rd.nestle.com (I.M.); 2 Department of Science and Technology, Food and Nutrition Research Institute, Taguig City 1631, Philippines; iangelesagdeppa@yahoo.com.ph (I.A.-A.); mar_v_c@yahoo.com (M.V.C.)

**Keywords:** diet diversity, micronutrient adequacy, micronutrient fortification, Filipino schoolchildren

## Abstract

Previous studies have shown that the dietary diversity of young Filipino children to be limited and that the prevalence of nutrient inadequacies is high. This study extends the current knowledge to examine the relationship between diet diversity and the probability of adequacy of micronutrients among Filipino schoolchildren (aged 6 to 12 years), by the wealth status and dwelling location. The dietary intake data were collected using a single 24-h recall from 6460 children in the Filipino National Nutrition Survey 2013. The diet diversity score (DDS) and the probability of adequacies (PA) of 11 micronutrients were calculated, and further stratified by socio-economic status (SES) and dwelling location. The diet diversity was generally low (mean DDS = 4 out of 9). Children from the lowest SES, and living in rural areas, tended to have a lower DDS. Children with a DDS of 1 were likely to be inadequate in all 11 micronutrients. The higher DDS (≥6) was associated with higher PAs for the B vitamins but not for calcium, folate, iron, vitamin A and to large extent, vitamin C. This suggests that it was difficult for this population to achieve adequacy in these 5 micronutrients. More rigorous research on the topic is needed. Better access to nutrient-rich or fortified staple foods, in tandem with increased education on the importance of dietary diversity, are potential strategies to support children in achieving adequate micronutrient intakes.

## 1. Introduction

Malnutrition, including underweight and stunting, is still a major public health concern in the Philippines. The prevalence of underweight Filipino children aged 5 to 10 years is 31.2%, stunting 31.1%, and wasting/thinness 8.4%. However, overweight children also exist in this age group (8.6% nationally), with a higher prevalence in urban than rural areas (13.0% versus 5.1%) [1]. Malnutrition is partially attributable to poor dietary quality, with 2 out of 3 Filipino households experiencing food insecurity [2]. The results of the latest Filipino National Nutrition Survey (2013) demonstrates inadequate nutrient intakes in schoolchildren, particularly with respect to poor intakes of calcium, iron, phosphorus, vitamin A, vitamin C and some B vitamins [3]. These nutrients are vital for growth and development in this young, vulnerable population [4]. Moreover, higher nutrient inadequacies were observed in Filipino schoolchildren from a rural dwelling location or from a poorer wealth status, which may be due to the limited access to affordable, fresh food [3]. On the other hand, it has also been reported that a small proportion (between 3–16%) of schoolchildren 6 to 12 years have excessive fat, protein and carbohydrate intakes in the Philippines, particularly those from the higher socioeconomic groups [3]. While little is known about nutrition transition among Filipino children, a previous study on the double burden of malnutrition in the Philippines between 1978 to 2003 has shown that although the mean food intake per capita in Filipino households did not change overtime, the mean energy intake per capita increased from 1804 kcal/day to 1905 kcal/day over the 25 year period. Furthermore, the consumption of meat, meat products and poultry; sugars and syrups; fats and oils increased while fruit and vegetable consumption reduced over this period. Rice remained as the country’s top staple food and source of energy [5].

While there has been progress in addressing undernutrition in the Philippines, it is still a problem of far greater magnitude than overnutrition is among children [5]. Limited dietary diversity can contribute to inadequate micronutrient intakes. Dietary diversity scores (DDS) have been implemented in a number of developing countries to evaluate dietary diversity and micronutrient adequacy in children and adults [6,7,8,9]. Higher dietary diversity has been associated with reduced stunting. Rah et al. identified that children (aged < 5years) in the higher diet diversity group were 30% less likely to be stunted compared to children from the lower diet diversity group [6]. A higher DDS has also been associated with increased micronutrient intakes [9,10]. A DDS score was applied to 9 food groups in a cross-sectional survey of children and women in rural Bangladesh. The prevalence of micronutrient inadequacy was 43% in children and 26% in women, which was primarily explained by diets low in energy and little diversity of foods [10]. Furthermore, a study in Ghanian children demonstrated significant improvement of nutrient intakes with an increasing DDS score. Moreover, the DDS was associated with impaired growth, wherein children with a lower DDS presented lower weight- and height-for-age and weight-for-length scores [9].

Kennedy et al. demonstrated the use of DDS, based on 10 food groups, to predict the probability of micronutrient adequacy in 2–5 year old Filipino children [7]. This group of young children consumed a relatively limited diet of four or five of food groups per day, with the mean probability of adequacy (MPA) of 11 micronutrients being 33% [7].

While the nutrient intakes of Filipino schoolchildren have been previously described [3], the dietary diversity, based on food group consumption of Filipino schoolchildren and its relationship with the probability of adequacy of key micronutrients, is unknown. Furthermore, the impact of socio-economic status (SES) and dwelling location on dietary diversity in a Filipino population has not been explored. Therefore, the aim of this study was to characterise dietary diversity among school age children (6–12y), and examine its relationship with the probability of adequacy of 11 micronutrients by SES and dwelling location (urban versus rural). The results of this study could inform further research and provide policy makers evidence on the potential association between dietary diversity and micronutrient intakes in Filipino schoolchildren.

## 2. Materials and Methods

### 2.1. Study Population

The dietary intake data from 6460 children, aged 6 to 12 years, who participated in the 2013 National Nutrition Survey (NNS) were used in the current analyses. The details of the NNS have been previously outlined [1]. In brief, the 2013 NNS was a cross-sectional, population-based survey that collected information on the health and nutritional status of the Filipino population. Filipino households (*n* = 35,825) were sampled with a response rate of 91%. The Ethics Committee of Food Nutrition Research Institute (FNRI) approved the survey protocol and data collection instruments. All surveyed households provided informed consent prior to participation.

### 2.2. Data Collection

Trained, registered dietitians, conducted face-to-face 24-h dietary recalls with a parent or caregiver of each child during household visits, wherein the dietician recorded all food and beverages that the child consumed the previous day. A first 24-h recall was performed for all children and a second 24-h recall was repeated in 50% of randomly selected households, typically 2 days after the first recall. Only the first 24-h recall was used in the current analysis. The amount of each food item or beverage was estimated using common household measures such as cups, tablespoons, by size or the number of pieces. The information was then converted to grams using a portion-to-weight list for common foods compiled by the FNRI or through weighing of the food samples. The nutrient composition of the data was cleaned, quality controlled and processed by the FNRI prior to analysis. This included reviewing all foods and drinks reported at the individual level to ensure that all the codes and quantities were entered correctly. The food and beverages consumed were converted to energy and nutrient intakes using the Filipino Food Composition Tables. Eleven micronutrients, as validated by Kennedy et al. [7] comprising calcium, thiamine, riboflavin, niacin, iron, vitamins A, C, B6, B12, folate and zinc were retained for the current analysis. The outliers were defined as intakes above the 99th percentile, as per Lopez-Olmedo et al. [11] and were replaced by the median value of the corresponding variable.

The 979 unique food items reported were categorised into 9 major food groups and further subgroups. The food grouping system was adapted from What We Eat in America Food Categories [12] and the US Feeding Infants and Toddlers Study (FITS) adjusted for the Philippines local food culture [13].

### 2.3. Diet Diversity Score (DDS)

In the current analysis, the aforementioned food categories were further assigned to 10 DDS food groups (cereals and tubers; meat, poultry and fish; dairy; eggs; pulses and nuts; vitamin A-rich fruits and vegetables; other fruit; other vegetables; oils and fats; and other). This DDS food grouping system was defined based the outcome of a Food and Agriculture Organization (FAO) workshop on the validation methods for dietary diversity [14]. Kennedy et al. examined and validated the use of these 10 food groups to reflect diet diversity in Filipino children, aged between 2 and 5 years old [7]. In the current study, if a child consumed a minimum of 10 g of at least one food item belonging to a DDS food group, excluding oils and fats, he/she would receive a maximum score of 1 for that particular DDS group. Previous validation studies have demonstrated that applying a minimum food group consumption of 10 g provided a better representation of nutrient adequacy in children [7,15]. The DDS for each child was determined based on the sum of the number of individual DDS food groups consumed in their 24-h recall. The DDS ranged from a minimum score of 0 to a maximum of 9.

### 2.4. Probability of Adequacy (PA) of Micronutrients

An existing algorithm developed by Foote et al. (2004) was used to estimate a child’s probability of adequacy (PA) of a given micronutrient [16]. The PA is defined as the probability that a child’s nutrient intake is adequate on a single day [15]. The dietary requirement of the nutrient is assumed to follow a normal distribution, with an age and gender specific cut-off for the estimated average requirement (EAR) and standard deviation (SD). The PA is calculated based on the probability of the cumulative distribution function (CDF) multiplied by the difference of the estimated child intake and EAR, divided by the SD. For iron, as its distribution of requirement is skewed and therefore this equation was not applicable, its PA was derived from Tables 1–5 within the IOM manual for iron [17] as recommended by Kennedy and Nantel [14].

(1) $$PA_{i}=Prob.CDF\frac{estimated\;child\;intake_{i}-EAR_{i}}{SD_{i}}$$

(2) $$MPA=\frac{\sum PA_{i}}{N}$$

The PA of each nutrient was calculated for the total population, and further stratified by the socioeconomic status and dwelling location. The mean of the 11 PAs (MPA) was calculated to provide an estimate of the overall probability of adequacy of the key micronutrients of the sample population. All nutrient intakes were converted to milligrams per day (mg/d) prior to the analysis.

### 2.5. Statistical Analysis

The DDS, PA and MPA were calculated as described above. The cross-tabulations and χ2 statistics were applied to determine the associations between the gender, wealth status and dwelling locations. The associations between the PA for each nutrient, DDS, dwelling locations and SES were examined using regression models.

To identify if certain micronutrients were driving the MPA, a set of gradient boosting regressor models (GBMs) were applied, as predictors of probability of inadequacy (defined as 1-MPA) based in the micronutrient intake. The GBMs were preferred due to the skewness of the nutrient intake data. The model parameter optimization was performed by a grid search and internal cross validation (11 segments). To ensure the validity of the models, a random subsample was selected and the same model was applied to check for congruency. 

All calculations were performed in an Anaconda Python 3.6.7 environment running on a HP Z440 workstation in Windows 10. To perform model optimization and validation, the modules from Sklearn v.0.20.1 library were applied [18]. The data handling and manipulation was done using Pandas 0.24.1. For statistical analyses, the Scipy v.1.1.0 library was used [19].

## 3. Results

### 3.1. Descriptive Statistics

**Figure 1:**
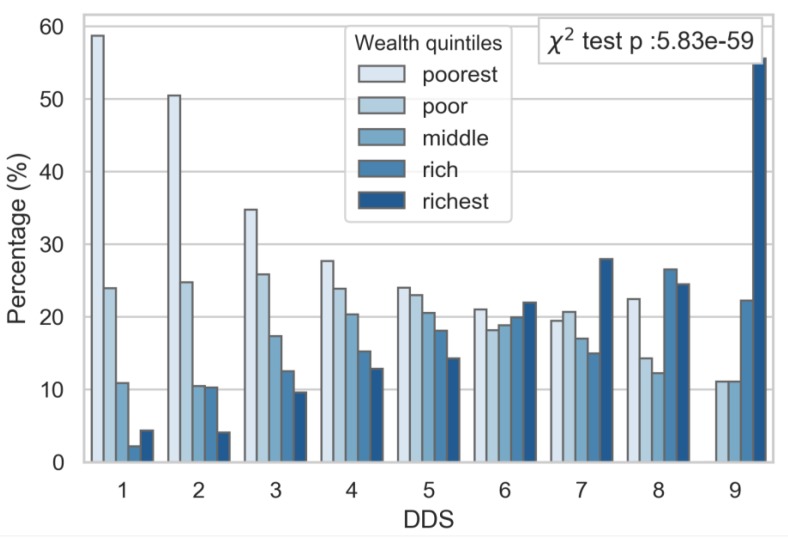
Percentage of children in each diet diversity score (DDS) group by socio-economic status.

**Figure 2:**
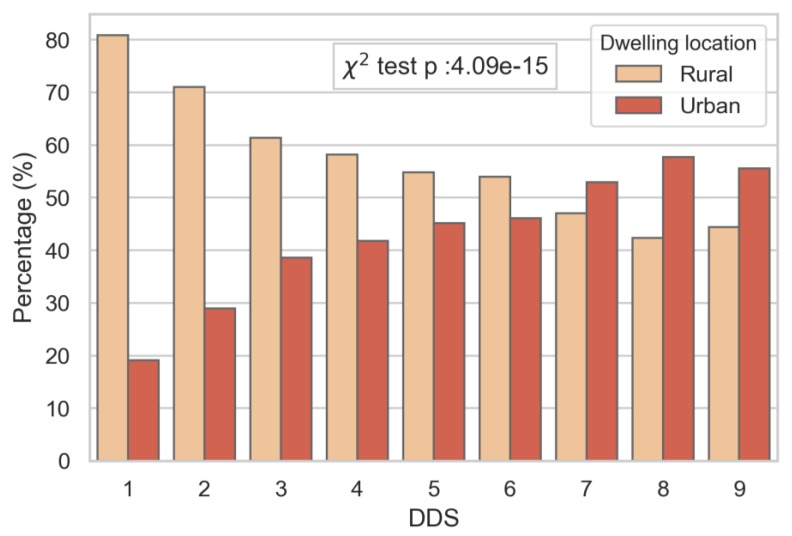
Percentage of children in each diet diversity score (DDS) group by dwelling locations (urban and rural).

A description of the sample population is shown in Table 1. The mean age was 9.61 years (SD: ±2. Half of the sample came from the poor and poorest socio-economic classes, and living in rural dwellings. The mean DDS was 4, with the majority of children consuming three to five food groups per day. The contingency table analysis showed significant relationships between the socio-economic status, dwelling location (Supplementary Figure S1) and DDS, independent of gender (data not shown). The socio-economic status was strongly associated with DDS; whereby those from the highest socio-economic group had the highest diet diversity (Figure 1), and those with lower DDS were predominantly from lower socio-economic groups. No children from the poorest SES group reached a DDS of nine. A similar trend was observed by dwelling locations, with a greater proportion of children from a rural location presenting a lower DDS (Figure 2).

**Figure 3:**
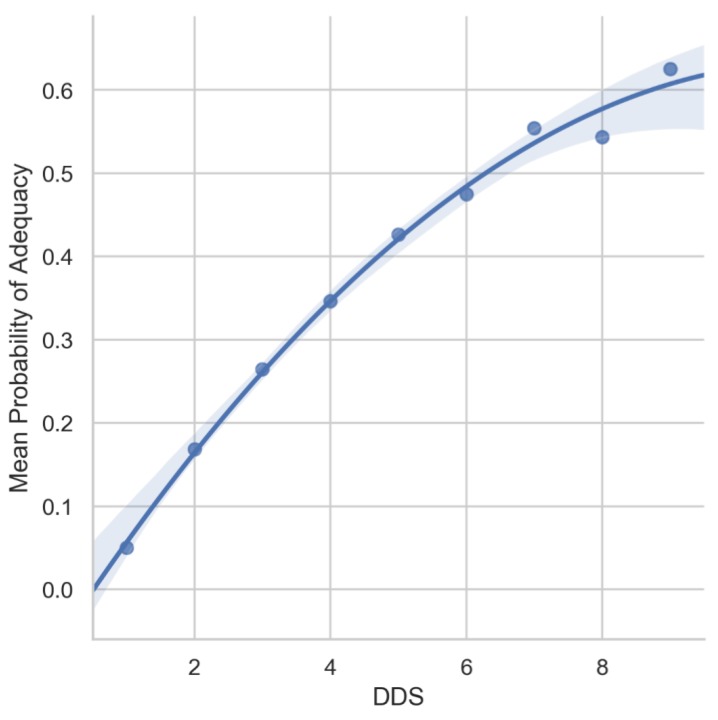
Mean probability of adequacy as the average of probability of adequacies (PA) of 11 micronutrients, presented by the diet diversity score (DDS) level.

Figure 3 illustrates the non-linear relationship between the DDS and MPA. The result showed that a DDS of 4 (the mean score of this population), the mean probability of adequacy across the 11 micronutrients was approximately 35%. Even at the highest DDS of 9, the mean probability of adequacy across the 11 micronutrients was only approximately 60%.

### 3.2. DDS and PA

The relationships between DDS and the probability of adequacy of each individual nutrient are summarized in Table 2. A PA score of 1 indicates the probability of adequacy of a nutrient was 100%, while 0 indicates the probability of being adequate was 0%. Children with a DDS of 1 were likely to be inadequate in all 11 micronutrients. The probability of adequacy of nutrients improved with increasing DDS. Even with relatively low DDS (e.g., DDS ≤ 4, the mean score of this population), children were likely to be adequate in vitamin B12, thiamine and vitamin B6. Children with a DDS of 9 had the highest number of nutrients with PA = 1 (100% probability of adequacy), however, for calcium, folate, vitamin A and vitamin C, the PA remained at 0.

### 3.3. DDS, PA, and Dwelling Locations

Table 3 illustrates the PA scores stratified by urban and rural areas. While the overall patterns of PA were similar between the two populations, some differences were observed. Children from urban areas were likely to reach 100% adequacy in riboflavin, thiamine and zinc with lower DDS scores compared to children from rural areas. For example, children from urban areas could reach 100% adequacy in zinc at a DDS of 7 while children from rural areas would need a DDS of 8 or 9 to reach full adequacy.

### 3.4. DDS, PA, and SES

The patterns of PA by DDS and SES are comparable to those by dwelling locations, although the differences between the poorest and the richest are more prominent. As shown in Table 4, at the highest DDS among the children from the poorest households, they were only adequate in 5 out of 11 micronutrients. Children from the richest households on the other hand, at a DDS of 9, were adequate in 7 of the 11 micronutrients. The majority of children from the poorest group had a DDS between 1 and 4, suggesting that these children were likely to be adequate in only 2 (Vitamin B12 and B6) out of 11 micronutrients. Furthermore, zinc intakes were related to SES, wherein 100% adequacy was achieved at a DDS of 6 in the richest children, but not in the poorest children.

## 4. Discussion

### 4.1. Diet Diversity among Filipino Children

The current study examined the relationship between diet diversity of Filipino school age children (6–12y) and their probability of adequacy of 11 micronutrients. The diet diversity was low with an average of four out of nine food groups per day. A previous study in younger Filipino children found similar findings on DDS [7], wherein diets were composed of limited foods, namely a large amount of refined rice and non-nutritious foods [13]. The current study demonstrates that older Filipino children also consume a relatively monotonous diet that lacks variety, and nutrient inadequacy is prevalent. Furthermore, this study confirms that characterising children by SES and dwelling locations are important when examining the relationships between DDS and PA, which has not been previously demonstrated.

### 4.2. Socio-Economic Status and Urban versus Rural Children

This study showed that nutrient adequacy was greater in those from higher socio-economic groups and urban areas than their lower SES and rural counterparts. Those from higher SES or urban areas met B-vitamin adequacy with relatively fewer food groups than rural or children from the lower SES. These results are in accordance with the recent study by Angeles-Agdeppa et al. [3] which found that micronutrient inadequacy was greater (thiamine, riboflavin, niacin, vitamin B6, phosphorus, zinc, and iron) in Filipino schoolchildren and adolescents from poorer families and living in rural areas. Interestingly, they found a higher prevalence of inadequacy for vitamin C (10–12 years), vitamin A (6–9 years) and folate (6–9 years) among urban children. However, no difference in the probability of adequacy of vitamin A and folate according to DDS groups by dwelling location was observed in the current study. Previous studies have suggested that children from lower SES/rural areas have greater consumption of vegetables and fruit (including fortified fruit juices) than urban children [3,20]. This may explain the higher iron and vitamin C intakes among rural children. The association between zinc and the PA gradient was greater for individuals in the urban areas. This suggests that it is more achievable for this subgroup of the population to reach zinc adequacy, and by consuming a lower number of food groups. 

### 4.3. Calcium, Folate, Iron, Vitamin A and Vitamin C Adequacies are Difficult to Meet

The literature suggests that Filipino children are most at risk of calcium, iron, vitamin C, folate, vitamin A, riboflavin, thiamine and phosphorus inadequacies [3]. This study suggests that it is difficult for Filipino schoolchildren to be adequate in all 11 micronutrients, a finding that has not been previously demonstrated in this population. Although increasing the number of food groups in the diet can improve nutrient adequacy to some extent [9], this study has shown that even with a diverse diet (e.g., DDS ≥ 7), the probability of adequacies for calcium, folate, vitamin A and vitamin C remained as zero. The simple view that increasing diversity of a diet can reduce inadequacy of nutrient intakes does not appear to hold true for all micronutrients. This is likely because the commonly consumed food sources are not rich enough in these nutrients, or the consumption of foods rich in calcium, folate, vitamin A and vitamin C is too low, or both. The potential solutions include significantly increasing the quantity of foods that are rich in these nutrients in the diet, to fortify or increase the level of fortification of commonly consumed foods from the different food groups. Indeed, for calcium for example, increased milk consumption would help close this nutrient gap. However, the previous National Nutrition Survey in the Philippines indicated that less than a third of school-age children consume any milk [21]. Therefore, the low intake of calcium may be attributed to the availability and affordability of milk, dairy products, or other food sources of calcium, such as fish, for those who are lactose intolerant [3,13]. Thus, an increased consumption of calcium-rich foods may help close this nutrient gap.

Increasing nutrient rich foods in the diet could increase the cost of diet, which may not be ideal for low-income families. A national food fortification programme could, therefore, be a more cost-effective and viable solution to address micronutrient inadequacy among poorer or rural households. A mandatory food fortification law of staple foods such as wheat flour, rice, cooking oil and sugar has existed since 2004 in the Philippines. However, a number of challenges have been reported to hinder its success [22]. Better access to fortified foods and a more enhanced, a widespread fortification programme to target staples as well as popular foodstuffs for children may remove some of these challenges. Indeed, a RCT conducted in the Philippines has demonstrated that a non-carbonated fortified juice drink with iron and zinc was effective in reducing the prevalence of anemia and improved the zinc status of children [23]. Similarly, a study demonstrated that fortified milk can improve the nutritional status of school children. This RCT identified a micronutrient fortified milk-based drink as effective in improving the micronutrient status of vitamin B2, vitamin B12 and red cell folate and in preventing a decline in hemoglobin levels, compared to an unfortified milk-based drink among South Indian children. [24]. A further study demonstrated the benefits of regular milk and fortified milk consumption among children in rural Vietnam. Both the weight-for-age and height-for-age significantly improved during 6 months of intervention among milk drinkers and fortified milk drinkers, and underweight and stunting dropped by 10% in these groups compared to the controlled group. Fortified milk consumption also showed improvements in physical and mental performance [25]. Nonetheless, an extensive fortification strategy with increased accessibility to nutrient-rich foods, combined with personalised nutrition education to children and their caregivers, are some of the potential strategies to improve the adequacy of multiple micronutrients among children in the Philippines.

### 4.4. Strengths and Limitations

This study has demonstrated that a diverse diet does not guarantee the adequacy of key micronutrients in Filipino children. Moreover, the patterns of micronutrient probability of adequacy can vary by dwelling locations and SES, even if children are eating the same number of food groups, which has not been previously described in this Filipino school-aged population. The large sample of children (n = 6460) from a nationally representative survey of the Philippines covering the age group 6 to 12 years is a strength of this study.

There are however, limitations to be highlighted. Only one day of the 24-h recall was used in the estimation of DDS and PAs and therefore cannot represent habitual food consumption or nutrient intakes. Consequently, self-reported bias such as under- or over-reporting is plausible among the sample. A further limitation is that most children had DDS scores between 3 and 5, and the number of children with DDS < 2 or DDS > 6 was low. There were more children in the high DDS groups from urban areas or higher SES than rural or poorer families. Therefore, readers should take caution when interpreting results on PAs for these higher DDS groups.

While previous studies have shown the strengths of using DDS as an indicator of diet diversity in developing countries, given the Philippines are going through a nutrition transition where the double burden of undernutrition and overweight exists, caution should be taken when categorising nutrient-dense and energy-dense foods into DDS groups. While the authors followed the DDS food groups suggested by the literature on younger Filipino children, an improvement for the future could be to increase the sensitivity of DDS groups to different types of nutrient-rich or fortified foods versus foods high in energy and low in micronutrients. Further research on nutrition transition in the Philippines as well as the relationship between diet diversity and nutrient intakes and overweight/obesity are needed, to support policymakers in addressing this emerging type of food insecurity.

## 5. Conclusions

This study provides novel insights into the dietary diversity of Filipino schoolchildren, and the associations between socioeconomic status, dwelling location and dietary diversity. The lack of dietary diversity is one of the reasons for the low probability of adequate intakes of micronutrients, particularly those from poorer households. The strategies such as wide-spread fortification, improved accessibility to fortified products, as well as tools to help increase a caregivers’ awareness and understanding on diet diversity and nutrient-rich foods may help to improve micronutrient intakes in the children population.

## Figures and Tables

**Table 1:** Descriptive statistics of the sample population (*n* = 6460).

**Age**	Mean: 9.6 years (SD: ±2)										
**Gender**	Girls: 51.5%; boys: 48.5%										
**Dwelling Location**	Urban: 41.5%, rural: 58.5%										
**Socio-Economic Status**	Richest		Rich		Middle		Poor			Poorest	
	12.9%		14.8%		17.9%		22.8%			28.9%	
**Diet Diversity Score**	Mean: 4.1 (SD: ±1.3)										
	1	2	3	4	5	6		7	8		9
	0.7%	8.3%	24.8%	29.9%	21.4%	10.0%		3.9%	0.8%		0.1%

**Table 2:** Distribution of probability of adequacy (PA) values of 11 micronutrients according to the diet diversity score (DDS).

DDS	Calcium	Folate	Iron	Niacin	Riboflavin	Thiamine	Vitamin B12	Vitamin B6	Vitamin C	Vitamin A	Zinc
**1**	0.0000	0.0000	0.0000	0.0000	0.0000	0.0000	0.0000	0.0000	0.0000	0.0000	0.0000
**2**	0.0000	0.0000	0.0000	0.0000	0.0000	0.0000	1.0000	0.0000	0.0000	0.0000	0.0000
**3**	0.0000	0.0000	0.0000	0.2500	0.0000	0.0000	1.0000	1.0000	0.0000	0.0000	0.0020
**4**	0.0000	0.0000	0.0000	0.9536	0.9940	1.0000	1.0000	1.0000	0.0000	0.0000	0.0880
**5**	0.0000	0.0000	0.0000	1.0000	1.0000	1.0000	1.0000	1.0000	0.0000	0.0000	0.6530
**6**	0.0000	0.0000	0.0000	1.0000	1.0000	1.0000	1.0000	1.0000	0.0000	0.0000	0.9389
**7**	0.0000	0.0000	0.0620	1.0000	1.0000	1.0000	1.0000	1.0000	0.0000	0.0000	1.0000
**8**	0.0000	0.0000	0.2360	1.0000	1.0000	1.0000	1.0000	1.0000	0.0000	0.0000	1.0000
**9**	0.0000	0.0000	1.0000	1.0000	1.0000	1.0000	1.0000	1.0000	0.0060	0.0000	1.0000

Probability of adequacy (PA) is the probability that a child’s nutrient intake is adequate on a single day. PA = 0 indicates the probability of being adequate of a particular nutrient is 0%; PA = 1 (shaded) indicates the probability of being adequate of a particular nutrient is 100%. The diet diversity score (DDS) refers to the number of food groups (out of 9) a child has consumed on a single day.

**Table 3:** Distribution of probability of adequacy (PA) values of 11 micronutrients according to the diet diversity score (DDS), stratified by rural and urban areas.

Dwelling	DDS	Calcium	Folate	Iron	Niacin	Riboflavin	Thiamine	Vitamin B12	Vitamin B6	Vitamin C	Vitamin A	Zinc
**Rural**	1	0.0000	0.0000	0.0000	0.0000	0.0000	0.0000	0.0000	0.0000	0.0000	0.0000	0.0000
	2	0.0000	0.0000	0.0000	0.0000	0.0000	0.0000	1.0000	0.0000	0.0000	0.0000	0.0000
	3	0.0000	0.0000	0.0000	0.0560	0.0000	0.0000	1.0000	1.0000	0.0000	0.0000	0.0000
	4	0.0000	0.0000	0.0000	0.8110	0.0000	1.0000	1.0000	1.0000	0.0000	0.0000	0.0220
	5	0.0000	0.0000	0.0000	1.0000	1.0000	1.0000	1.0000	1.0000	0.0000	0.0000	0.5150
	6	0.0000	0.0000	0.0000	1.0000	1.0000	1.0000	1.0000	1.0000	0.0000	0.0000	0.7540
	7	0.0000	0.0000	0.0010	1.0000	1.0000	1.0000	1.0000	1.0000	0.0000	0.0000	0.9893
	8	0.0000	0.0000	0.0880	1.0000	1.0000	1.0000	1.0000	1.0000	0.0000	0.0000	0.9998
	9	0.0000	0.0000	1.0000	1.0000	1.0000	1.0000	1.0000	1.0000	0.2120	0.0000	1.0000
**Urban**	1	0.0000	0.0000	0.0000	0.0000	0.0000	0.0000	0.0000	0.0000	0.0000	0.0000	0.0000
	2	0.0000	0.0000	0.0000	0.0030	0.0000	0.0000	1.0000	0.0000	0.0000	0.0000	0.0000
	3	0.0000	0.0000	0.0000	0.6830	0.0000	1.0000	1.0000	1.0000	0.0000	0.0000	0.0270
	4	0.0000	0.0000	0.0000	0.9987	1.0000	1.0000	1.0000	1.0000	0.0000	0.0000	0.4110
	5	0.0000	0.0000	0.0000	1.0000	1.0000	1.0000	1.0000	1.0000	0.0000	0.0000	0.8410
	6	0.0000	0.0000	0.0010	1.0000	1.0000	1.0000	1.0000	1.0000	0.0000	0.0000	0.9949
	7	0.0000	0.0000	0.4570	1.0000	1.0000	1.0000	1.0000	1.0000	0.0000	0.0000	1.0000
	8	0.0000	0.0000	0.5410	1.0000	1.0000	1.0000	1.0000	1.0000	0.0000	0.0000	1.0000
	9	0.0000	0.0000	0.3660	1.0000	1.0000	1.0000	1.0000	1.0000	0.0000	0.0000	1.0000

Probability of adequacy (PA) is the probability that a child’s nutrient intake is adequate on a single day. PA = 0 indicates the probability of being adequate of a particular nutrient is 0%; PA = 1 (shaded) indicates the probability of being adequate of a particular nutrient is 100%. Diet diversity score (DDS) refers to the number of food groups (out of 9) a child has consumed on a single day.

**Table 4:** Distribution of Probability of Adequacy values of 11 micronutrients according to the diet diversity score (DDS), stratified by socio-economic status (poorest and richest groups shown).

SES	DDS	Calcium	Folate	Iron	Niacin	Riboflavin	Thiamine	Vitamin B12	Vitamin B6	Vitamin C	Vitamin A	Zinc
**Poorest**	**1**	0.0000	0.0000	0.0000	0.0000	0.0000	0.0000	0.0000	0.0000	0.0000	0.0000	0.0000
	**2**	0.0000	0.0000	0.0000	0.0001	0.0000	0.0000	1.0000	0.0000	0.0000	0.0000	0.0000
	**3**	0.0000	0.0000	0.0000	0.0024	0.0000	0.0000	1.0000	1.0000	0.0000	0.0000	0.0000
	**4**	0.0000	0.0000	0.0000	0.6563	0.0000	0.0000	1.0000	1.0000	0.0000	0.0000	0.0021
	**5**	0.0000	0.0000	0.0000	1.0000	1.0000	1.0000	1.0000	1.0000	0.0000	0.0000	0.2042
	**6**	0.0000	0.0000	0.0000	1.0000	1.0000	1.0000	1.0000	1.0000	0.0000	0.0000	0.6348
	**7**	0.0000	0.0000	0.0005	1.0000	1.0000	1.0000	1.0000	1.0000	0.0000	0.0000	0.9403
	**8**	0.0000	0.0000	0.0134	1.0000	1.0000	1.0000	1.0000	1.0000	0.0000	0.0000	0.9885
	**9**											
**Richest**	**1**	0.0000	0.0000	0.0000	0.3470	0.0000	1.0000	0.0000	0.5000	0.0000	0.0000	0.2519
	**2**	0.0000	0.0000	0.0000	0.2193	0.0000	0.0000	1.0000	1.0000	0.0000	0.0000	0.0125
	**3**	0.0000	0.0000	0.0000	0.9897	1.0000	1.0000	1.0000	1.0000	0.0000	0.0000	0.3302
	**4**	0.0000	0.0000	0.0000	1.0000	1.0000	1.0000	1.0000	1.0000	0.0000	0.0000	0.8195
	**5**	0.0000	0.0000	0.0001	1.0000	1.0000	1.0000	1.0000	1.0000	0.0000	0.0000	0.9915
	**6**	0.0000	0.0000	0.0093	1.0000	1.0000	1.0000	1.0000	1.0000	0.0000	0.0000	1.0000
	**7**	0.0000	0.0000	0.2964	1.0000	1.0000	1.0000	1.0000	1.0000	0.0000	0.0000	1.0000
	**8**	0.0000	0.0000	0.9539	1.0000	1.0000	1.0000	1.0000	1.0000	0.0000	0.0000	1.0000
	**9**	0.0000	0.0000	1.0000	1.0000	1.0000	1.0000	1.0000	1.0000	0.1209	0.0000	1.0000

Probability of adequacy (PA) is the probability that a child’s nutrient intake is adequate on a single day. PA = 0 indicates the probability of being adequate of a particular nutrient is 0%; PA = 1 (shaded) indicates the probability of being adequate of a particular nutrient is 100%. Diet diversity score (DDS) refers to the number of food groups (out of 9) a child has consumed on a single day.

## Supplementary Materials

The following are available online at https://www.mdpi.com/2072-6643/11/9/2197/s1, Figure S1: Distribution of percentage of children per wealth status group according to dwelling locations.

## References

[1] Food and Nutrition Research Institute (2015). Philippine Nutrition Facts and Figures 2013. 8th National Nutrition Survey Overview.

[2] Food and Nutrition Research Institute (2016). 2015 Updating of the Nutritional Status of Filipino Children and Other Population Group: Food Security Survey.

[3] Angeles-Agdeppa I., Denney L., Toledo M.B., Obligar V.A., Jacquier E.F., Carriquiry A.L., Capanzana M.V. (2019). Inadequate nutrient intakes in filipino schoolchildren and adolescents are common among those from rural areas and poor families. Food Nutri Res..

[4] World Health Organization Early Child Development—Nutrition and the Early Years. https://www.who.int/topics/early-child-development/child-nutrition/en/.

[5] Pedro M.R.A., Benavides R.C., Barba C.V.C. (2006). Dietary Changes and Their Health Implications in the Philippines, in The Double Burden of Malnutrition: Case Studies from Six Developing Countries.

[6] Rah J.H., Akhter N., Semba R.D., De Pee S., Bloem M.W., Campbell A.A., Kraemer K. (2010). Low dietary diversity is a predictor of child stunting in rural Bangladesh. Eur. J. Clin. Nutr..

[7] Kennedy G.L., Pedro M.R., Seghieri C., Nantel G., Brouwer I. (2007). Dietary diversity score is a useful indicator of micronutrient intake in non-breast-feeding Filipino children. J. Nutr..

[8] Arimond M., Ruel M.T. (2004). Dietary diversity is associated with child. Nutritional status: Evidence from 11 demographic and health surveys. J. Nutr..

[9] Nti C. (2011). Dietary diversity is associated with nutrient intakes and nutritional status of children in Ghana. Asian J. Med Sci..

[10] Arsenault J.E., Yakes E.A., Islam M.M., Hossain M.B., Ahmed T., Hotz C., Brown K.H. (2013). Very low adequacy of micronutrient intakes by young children and women in rural Bangladesh is primarily explained by low food intake and limited diversity. J. Nutr..

[11] López-Olmedo N., Carriquiry A.L., Rodríguez-Ramírez S., Ramírez-Silva I., Espinosa-Montero J., Hernández-Barrera L., Rivera J. (2016). A usual intake of added sugars and saturated fats is high while dietary fiber is low in the Mexican population. J. Nutr..

[12] USDA What We Eat in America Food Categories. https://www.ars.usda.gov/ARSUserFiles/80400530/pdf/1314/food_category_list.pdf.

[13] Denney L., Angeles-Agdeppa I., Capanzana M., Toledo M., Donohue J., Carriquiry A. (2018). Nutrient intakes and food sources of Filipino infants, toddlers and young children are inadequate: Findings from the national nutrition survey 2013. Nutrients.

[14] Kennedy G.L., Nantel G. (2006). Basic Guidelines for Validation of a Simple Dietary Diversity Score as an Indicator of Dietary Nutrient Adequacy for Non-Breastfeeding Children 2–6 Years.

[15] Daniels M.C., Adair L.S., Popkin B.M., Truong Y.K. (2007). Dietary diversity scores can be improved through the use of portion requirements: An analysis in young Filipino children. Eur. J. Clin. Nutr..

[16] Foote J.A., Murphy S.P., Wilkens L.R., Basiotis P.P., Carlson A. (2004). Dietary variety increases the probability of nutrient adequacy among adults. J. Nutr..

[17] Institute of Medicine (2001). Dietary Reference Intakes for Vitamin A, Vitamin K, Arsenic, Boron, Chromium, Copper, Iodine, Iron, Manganese, Molybdenum, Nickel, Silicon, Vanadium and Zinc.

[18] Pedregosa F., Varoquaux G., Gramfort A., Michel V., Thirion B., Grisel O., Vanderplas J. (2011). Scikit-learn: Machine learning in python. J. Mach. Learn. Res..

[19] Jones E., Travis E., Peterson P. SciPy: Open Source Scientific Tools for Python; 2001. http://www.scipy.org/.

[20] Santos L.P., Assunção M.C.F., Matijasevich A., Santos I.S., Barros A.J. (2016). Dietary intake patterns of children aged 6 years and their association with socioeconomic and demographic characteristics, early feeding practices and body mass index. BMC Public Health.

[21] Angeles-Agdeppa I.G.G., Constantino M. (2016). Evaluation of calcium intakes of young children in the Philippines as a result of the 2008 national nutrition survey. Philipp. J. Sci..

[22] Vega M.L.A. (2000). Fortification Efforts in the Philippines: Successes and Challenges.

[23] Angeles-Agdeppa I., Magsadia C.R., Capanzana M.V. (2011). Fortified juice drink improved iron and zinc status of schoolchildren. Asia Pac. J. Clin. Nutr..

[24] Kuriyan R., Thankachan P., Selvam S., Pauline M., Srinivasan K., Kamath-Jha S., Kurpad A.V. (2016). The effects of regular consumption of a multiple micronutrient fortified milk beverage on the micronutrient status of school children and on their mental and physical performance. Clin. Nutr..

[25] Lien D.T.K., Nhung B.T., Khan N.C., Hop L.T., Nga N.T.Q., Hung N.T., te Biesebeke R. (2009). Impact of milk consumption on performance and health of primary school children in rural Vietnam. Asia Pac. J. Clin. Nutr..

