# Supplementary material for: Diet Diversity and Micronutrient Adequacy among Filipino School-Age Children
Source: Nutrients. 2019 Sep 12;11(9):2197. doi: 10.3390/nu11092197 (PMC6770711; doi:10.3390/nu11092197)

**Supplementary Materials**

Figure S1. Distribution of percentage of children per wealth status group according to dwelling locations.

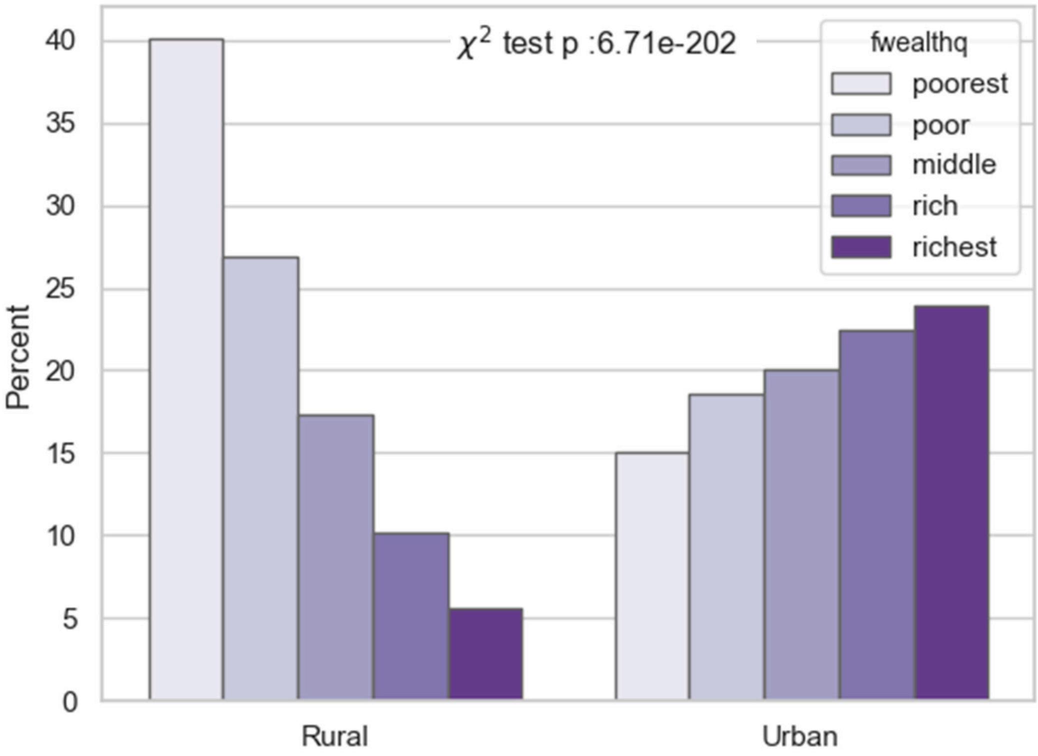

Supplement: Supplementary file 1 [file nutrients-11-02197-s001.pdf]
